# Increase in Postoperative Body Mass Index in Patients After Total Knee Arthroplasty

**DOI:** 10.7759/cureus.46203

**Published:** 2023-09-29

**Authors:** Maximiliano Barahona, Macarena A Barahona, Tomas Navarro, Pablo Chamorro, Anselmo Alegría, Martin Guzman, Miguel J Palet

**Affiliations:** 1 Orthopaedics Department, Hospital Clinico Universidad de Chile, Santiago, CHL

**Keywords:** knee osteoarthritis, body mass index (bmi), functional evaluation, patient-reported outcome, obesity, total knee replacement (tkr), primary total knee arthroplasty

## Abstract

Objectives

The aim of this is to investigate the changes in body mass index (BMI) following knee arthroplasty and to evaluate their impact on patient-reported outcomes and functional evaluations.

Methods

This observational study included 90 patients who underwent total knee arthroplasty (TKA) and were followed up for a median period of 2.6 years. BMI measurements were recorded before and after surgery, and patient-reported outcomes and functional evaluations were assessed using standardized scales and tests.

Results

Following TKA, BMI increased statistically significantly (Wilcoxon signed-rank test, p < 0.000). In addition, half of the patients experienced an increase in BMI, with 32% moving up in their BMI category. However, there were no clinically significant differences in patient-reported outcomes or functional evaluations between the group that gained BMI and the group that maintained or lost BMI.

Conclusion

This study reveals that patients tend to have increased BMI following TKA. However, these BMI changes do not significantly impact patient-reported outcomes or functional evaluations. It underscores the importance of patient education regarding healthy lifestyle habits, including diet and physical activity, to address postoperative weight gain effectively.

## Introduction

Obesity is a widespread disease, affecting more than 600 million individuals worldwide and carrying a significant burden of cardiovascular, pulmonary, and functional disorders [1]. Body mass index (BMI) serves as an indicator of a patient's nutritional status, with obesity generally defined as a BMI greater than 30. However, different values define overweight and obesity in elderly patients [2].

Knee osteoarthritis, a condition that impairs joint function and diminishes the quality of life, is also rising [3]. Obesity and osteoarthritis are closely intertwined, with studies indicating that individuals with a high BMI have a four to five times higher risk of developing knee osteoarthritis [4,5]. Exercise is critical to treat obesity and knee osteoarthritis [6,7], underscoring the importance of knee functionality. Consequently, orthopedic surgeons frequently encounter obese or overweight patients with end-stage knee osteoarthritis who require total knee arthroplasty (TKA). Understanding the challenges associated with these patients is paramount, as many obese patients attribute their knee problems to avoiding exercise.

TKA is a well-established and effective intervention for alleviating pain and improving the quality of life in patients with knee osteoarthritis [8]. Patient’s motivation after TKA strongly influences the likelihood of patients returning to sports activities [9]. However, obesity and overweight increase the risk of surgical site infections, prolong hospital stays, and lead to higher readmission rates [10,11]. Studies have also demonstrated that obese patients have poorer post-operative EQ-5D and Oxford scores. However, the level of improvement is comparable to that of non-obese patients six months after surgery [12].

This study aims to investigate the changes in BMI following knee arthroplasty and to evaluate their impact on patient-reported outcomes and functional evaluations. We hypothesize that patients will maintain their BMI after surgery, and any changes in BMI will not significantly affect the patient-reported outcome measures (PROMs).

## Materials and methods

An observational study was designed with approval from the ethics committee and was conducted in accordance with the declaration of Helsinki. In a previous study [13], the hospital records were reviewed to identify patients who underwent TKA with a cruciate retaining (CR) model and anterior stabilized insert between 2018 and 2020. Patients with postoperative infection (n=1), instability (n=1) of the prosthesis, prostheses with a posterior stabilized insert, revisions, and unicompartmental arthroplasty were excluded. A total of 105 patients were contacted and evaluated in person after excluding 15 patients whose preoperative weight and height were recorded only in the anesthesia sheet, which relied on verbal reporting by the patients rather than objective documentation like the nursing sheet.

The in-person evaluations were conducted by a physiotherapist who objectively measured the height and weight to calculate the BMI. Patients aged 65 years or older at the time of surgery were classified according to elderly parameters: 28 kg/m² or less as "normal," 28.1 to 31.9 kg/m² as "overweight," 32.0 to 36.99 kg/m² as "obese," and 37 kg/m² or greater as "severely obese." Patients below 65 years at the time of surgery were classified as follows: less than 25 kg/m² as "normal," 25.1 to 29.9 kg/m² as "overweight," 30.0 to 34.9 kg/m² as "obese," and above 35.0 kg/m² as "severely obese."

During the in-person evaluations, the validated Spanish versions of the Goodman Satisfaction Scale, as well as WOMAC, KUJALA, and KOOS quality-of-life scales were administered. Joint range of motion using a goniometer, algometry, the "up and go" test, and the "sit-up" test were also recorded.

For the statistical analysis, the patients were divided into two groups. Those with an increase of up to 1 kg/m² or a decrease in their BMI were categorized as the "same or lower" group, while those with an increase greater than 1 kg/m² were labeled as the "up" group. The comparisons of continuous variables between the groups were performed using the non-parametric median test, while categorical variables were compared using Fischer's exact test.

Furthermore, the preoperative BMI median was compared with the postoperative BMI using a paired median test, and the frequency (total and percentage) of patients who moved up or down in BMI categories was calculated. The significance level used for the analyses was 0.05, and the statistical software STATA Version 17.0 (StataCorp, College Station, TX) was used.

## Results

A total of 90 patients were included in the study. The median age of the participants was 67 years (range: 47 to 88; interquartile range: 62 to 71). The median follow-up period was 2.6 years (range: 1.1 to 5.1; interquartile range: 1.7 to 3.7).

The median BMI before surgery was 28.68 kg/m² (range: 21 to 39; interquartile range: 26 to 32), while the median BMI after surgery was 30.75 kg/m² (range: 21 to 44; interquartile range: 28 to 33). The difference in BMI before and after surgery was found to be statistically significant (Wilcoxon signed-rank test, p < 0.000), and this significance remained even when dichotomized by age (65 years old) (Table 1).

**Table 1:** Comparison of BMI median before and after surgery for patients aged above and below 65 years. *Median test

Age	N	BMI before surgery (kg/m^2^) (range; IQR)	BMI after surgery (kg/m^2^) (range; IQR)	P-value*
Less than 65	40	29 (21 to 39; 27 to 31)	31 (25 to 43; 29 to 33)	<0.0000
Above 65	50	29 (22 to 38; 25 to 32)	30 (21 to 44; 26 to 32)	0.0035

Out of the total patients, 45 (50%) patients were classified as “same or lower” BMI, while 45 (50%) had an increase in BMI and were classified as the “up” group. The “same or lower” group had a median change of -0.37 kg/m² (range: -3.70 to 0.94; interquartile range: -1.15 to 0.39). On the other hand, those in the “up” group had a median change of 3.08 kg/m² (range: 1.02 to 8.54; interquartile range: 2.07 to 4.93). The only variable that showed a statistically significant difference between these two groups was the follow-up time. While there was a tendency for a higher proportion of female and younger patients in the “up” group, this difference was not statistically significant. Neither patient-reported outcomes nor functional evaluations showed any clinically relevant differences between groups (Table 2).

**Table 2:** Comparison of patient-reported outcomes and functional evaluations between the “up” and “same or lower” groups. The values are given as n (%) or as mean (range; interquartile range) KOOS QL, Knee injury and Osteoarthritis Outcome Score quality of life; WOMAC, Western Ontario and McMaster Universities Osteoarthritis

	Same or Lower	Up	P-value
Age	67 (47 to 88; 62 to 72)	64 (47 to 78; 60 to 70)	0.1026
Gender			
Male	21 (58.33%)	15 (41.67%)	0.2820
Female	24 (44.44%)	30 (55.56%)	
Pain WOMAC	2 (0 to 14; 0 to 4)	2 (0 to 15; 1 to 5)	0.5803
Stiffness WOMAC	1 (0 to 8; 0 to 1)	1 (0 to 5; 0 to 1)	0.1533
Function WOMAC	6 (0 to 51; 4 to 15)	9 (0 to 55; 5 to 22)	0.1133
Goodman A	100 (50 to 100; 87.5 to 100)	93.8 (0 to 100; 81.25 to 100)	0.0917
Goodman B			
Much better	40 (51.28%)	38 (48.72%)	0.7580
Same or worse	5 (41.67%)	7 (58.33%)	
Kujala	78 (29 to 100; 66 to 90)	72 (32 to 96; 63 to 78)	0.0960
KOOS QL	68.75 (5 to 100; 50 to 87.5)	56.25 (12.5 to 100; 43.75 to 75)	0.2092
Range of motion	120 (91 to 147; 107 to 126)	114 (90 to 143; 108 to 122)	0.1708
Flexion	113 (95 to 135; 106 to 121)	111 (90 to 129; 106 to 115)	0.1640
Extension	4 (-12 to 12; 0 to 6)	2 (-2 to 16; 0 to 7)	0.4618
Sit-up test	13 (3 to 21; 11 to 14)	14 (7 to 27) 12 to 15)	0.2296
Up-go test	10 (6 to 33; 8 to 11)	9 (6 to 23; 8 to 11)	0.7354
Follow up	2.6 (1.13 to 4.46; 2.22 to 2.91)	2.9 (1.02 to 5.1; 1.61 to 3.84)	0.0358

Among the patients who were initially classified as "overweight," "obese," or "severely obese" (n = 64), four (6.25%) patients moved to a lower BMI classification after the surgery. Conversely, among the patients initially classified as "normal," "overweight," or "obese" (n = 86), 27 (32%) patients moved to a higher BMI classification.

Out of the 26 patients initially classified as "normal," 16 (61.53%) patients remained in the "normal" category, nine (34.61%) patients moved up to the "overweight" category, and one (3.84%) patient moved up to the "obese" category. Among the 37 patients initially classified as "overweight," 23 (62.16%) patients remained in the "overweight" category, one patient moved down to the "normal" category, 12 patients moved up to the "obese" category, and one patient moved up to the "severely obese" category. Of the 16 patients initially classified as "obese", nine of them remained in the same category, three moved down to the "overweight" category, and four patients moved up to the "severely obese" category. All four patients initially classified as "severely obese" remained in the same category (Table 3).

**Table 3:** Comparison of nutritional status based on pre-surgery BMI with last follow-up BMI

Preoperative nutritional status		Postoperative nutritional status			
		Normal	Overweight	Obese	Severely Obese
Normal	26	16	9	1	0
Overweight	37	1	23	12	1
Obese	23	0	3	16	4
Severely obese	4	0	0	0	4

## Discussion

The primary finding of this study is that patients who underwent TKA tend to experience an upward trend in BMI, particularly noticeable in patients under 65 years of age. The global average BMI increased by 2.07 kg/m2, and 27 (30%) patients moved up one level in their BMI classification. This study showed that the nutritional status of patients does not change just by achieving a functional improvement through joint replacement. Patients should be educated to adopt healthier habits, maintain a healthy diet, and perform physical activity, especially those who were overweight or obese before surgery.

A previous study reported a similar upward trend, reaching an increase in BMI category in 14-25% of the included patients, and only 65-68% maintained their BMI category [14]. However, there are also reports of early weight loss in a subset of patients. Teichtahl et al. [15] found that 37% of patients experienced weight loss in the first six months postoperatively, while Duchman et al. [16] observed weight loss in 11% of patients at a follow-up of 4.8 years, with an average loss of only 1.47 kg. Notably, the decrease in BMI seems to predominantly occur in the subgroups of obese and severely obese patients, whereas the overall trend is toward maintenance or increase [14].

In our study, half of the patients gained BMI, and 32% of the cohort went up in their BMI category. The “up” group showed a higher proportion of female and younger patients, but no statistically significant difference was found. Importantly, there were no statistically significant differences in functional evaluations between this group and the group that experienced an increase in BMI, except for a difference in follow-up time (2.6 vs. 2.9 years, p=0.0358).

Weight loss before surgery has been suggested to have beneficial effects, reducing complications and hospitalization length in the immediate postoperative period [17]. Numerous studies have associated a BMI of >30 with an increased risk of complications [18], and worse functional outcomes, pain, and dissatisfaction [19]. However, some studies have shown no significant difference in patient-reported outcomes when comparing normal-weight individuals with obese patients [20], or only a slower progression with similar long-term functional results [21]. Furthermore, Ponnusamy et al. [22] demonstrated that TKA was cost-effective regardless of BMI, thanks to the functional results achieved after surgery. Our study aligns with these findings, suggesting that the magnitude of weight gain may not be sufficient to hinder functional outcomes achieved by TKA. However, this weight gain after TKA has been linked to an increased rate of non-septic loosening of the tibial component [23]; therefore, it is crucial to prevent it and take measures to ensure it does not happen.

The tendency to maintain or gain weight postoperatively can be attributed to various factors such as psychological-behavioral aspects, social factors, changes in activity levels, or diet. For example, a prospective series demonstrated a 28% prevalence of anxiety symptoms and a 20% prevalence of depressive symptoms in the first postoperative year. It was also observed that although 31% of patients expected to lose weight, only 6.9% actually achieved weight loss, indicating that the trend continues despite the patient's intentions [14]. Additionally, preoperative weight loss did not correlate with postoperative weight loss, which is consistent with findings from other studies [24]. It is crucial to actively investigate and address these factors postoperatively to control weight gain.

The inclusion of nutritionists in the perioperative management protocol could be a subject of future study. Recently, Ueyama et al. [25] reported that supplementation with essential amino acid enhanced quadriceps muscle function, similar to what Dreyer et al. [26] and Muyskens et al. [27] reported years before. Moreover, Torchia et al. [28] reported that the screening of malnutrition before surgery was cost-effective, with a focus on preventing infection. These data suggest that the burden is higher, as it could also prevent weight gain and improve muscle strength, ultimately leading to an increased satisfaction rate after TKA. Moreover, four studies have demonstrated that patients undergoing TKA experience increased mortality for any cause after 12-15 years when compared to the general population [29-32]. The main attributed cause of this elevated risk is cardiovascular problems, which have been linked to osteoarthritis [29]. Obesity and cardiovascular disease are closely related, and thus the long-term impact of the gained BMI should be taken seriously [33,34].

One limitation of this study is the heterogeneity in the follow-up period, which may have influenced the timing of weight changes postoperatively. The varying lengths of follow-up could have contributed to differences in weight gain or loss in some cases; nevertheless, no statistically significant difference was found in this regard. Additionally, the sample size is another limitation. Although the study included enough patients to draw meaningful conclusions, a larger sample size could have provided more statistical power and precision. However, it is important to note that despite this limitation, the study's findings are consistent with previous research, supporting the robustness of the findings.

## Conclusions

In this series, with a minimum follow-up of one year, half of the patients had increased BMI in at least one unit, and 32% went up in their BMI category following knee arthroplasty. Therefore, it is necessary to include education on healthy eating and anxiety management and to encourage physical activity in the follow-up of these patients.
